# Impaired Cognitive Control of Emotional Conflict in Trait Anxiety: A Preliminary Study Based on Clinical and Non-Clinical Individuals

**DOI:** 10.3389/fpsyt.2018.00120

**Published:** 2018-04-06

**Authors:** Yongju Yu, Chenggang Jiang, Haiyan Xu, Qian Yang, Jiawen Li, Yuanyuan Xu, Wenjun Xiang, Li Peng, Botao Liu, Fang Lv, Min Li

**Affiliations:** ^1^Department of Military Psychology, School of Psychology, Third Military Medical University, Chongqing, China; ^2^Daping Hospital, Third Military Medical University, Chongqing, China; ^3^School of Nursing, Third Military Medical University, Chongqing, China

**Keywords:** trait anxiety, conflict detection, conflict resolution, conflict adaptation effect, generalized anxiety disorder

## Abstract

**Background:**

It has been observed that trait anxiety easily leads to conflict maladaptation under conflict circumstances. However, it remains unclear whether the precise neural mechanisms underlying the effects of high trait anxiety (HTA) on cognitive control are consistent in high trait anxious individuals, with and without anxiety disorders.

**Methods:**

The present study recruited 29 healthy volunteers with low trait anxiety (LTA), 37 healthy volunteers with HTA, and 23 patients with generalized anxiety disorder (GAD). All participants completed demographic information and self-report measures of trait anxiety and depression. Then, they performed the emotional flanker task with event-related potentials (ERPs) recorded.

**Results:**

Behavioral data manifested that, relative to LTA individuals, GAD patients displayed prolonged response times and increased error rates, while HTA individuals showed intact response times and accuracies. Event-related potential (ERP) data revealed that HTA individuals exhibited a trend toward more negative N2 amplitudes for conflict detection. By contrast, both HTA and GAD individuals displayed decreased P3 amplitudes for conflict resolution. ERP results indicated that both HTA and GAD individuals exhibited conflict maladaptation on the N2 amplitude. Correlation analyses also showed that the increased anxiety symptoms were associated with longer reaction times, more error rates, lower P3 amplitudes, and more perturbations in conflict adaptation on reaction times and N2 amplitudes.

**Conclusion:**

Our results demonstrated a severely impaired cognitive control in GAD patients while a moderately impaired cognitive control in HTA individuals. Trait anxiety can indeed serve as a predominant factor at the onset and in the maintenance of GAD. Therefore, the trait anxiety reducing strategies may provide significant therapeutic gains.

## Introduction

Increasing evidence has demonstrated that trait anxiety is related to impaired executive control of attention (1). The attentional control theory (ACT) proposed that anxiety is closely related to cognitive deficits (2), which makes it difficult for anxious individuals to efficiently inhibit distraction information. Therefore, anxiety has been considered to be able to inhibit attention, and it may be harder for trait anxious individuals to suppress threat-related irrelevant stimuli (2, 3). These deficits primarily affect processing efficiency, without adverse effects on performance effectiveness (1). Thus, in some cases participants with high anxiety show no greater evidence of disrupted attentional control behaviorally, but need to use more cognitive resources to perform at a level-standard relative to persons with low anxiety. These viewpoints, however, have not been systematically tested.

The face flanker paradigm allows for the efficient investigation of trait anxious individuals’ patterns of cognitive control, thereby illuminating how attention allocation is impacted by interactions between the target and distractor (4, 5). Reaction time interference by emotionally incongruent stimuli was observed in almost every individual (6, 7). That is, participants exhibit faster response speed when the distractor expressions are identical with the target expression. A large number of studies also showed that the emotional conflict generated by the previous incongruent trial can activate a regulatory mechanism which helps individuals to improve emotional conflict regulation on the current incongruent trial (8–10). Therefore, task performance was optimized. Likewise, performance on postcongruent congruent trials is often superior to that on postincongruent congruent trials. This across-trial effect has been termed as “emotional conflict adaptation” (11).

Event-related potential (ERP) studies have found that, in the conflict control processing, N2 and P3 components are associated with conflict detection and conflict resolution, respectively (12, 13). The conflict N2 component is a negative deflection peaking at about 200–300 ms after stimulus onset. It is derived from the anterior cingulate cortex and serves as an indicator of response conflict (10). It has been demonstrated that the N2 component on incompatible trials is larger than that on compatible trials (14, 15). When participants are attending more to flanker information than target information, a larger N2 amplitude will be elicited (16, 17). Empirical research found that, compared to healthy individuals, patients with generalized anxiety disorder (GAD) showed decreased N2 amplitudes for conflict adaptation in non-emotional flanker task that may be influenced by compensatory activity (18).

The P3 component is a positive-going ERP that peaks approximately 300–500 ms after stimulus presentation which serves as a marker of the active suppression of a motor response (i.e., conflict resolution) (19, 20). Most studies assume the flanker P3 to be functionally similar to the P3a (21, 22), reflecting activation in prefrontal brain (23). Research suggested that the P3 component elicited by stimulus conflict is larger for incongruent trials than that for congruent ones (13, 24) and proposed that the larger P3 amplitude elicited by incongruent trials is related to a more careful assessment of the stimulus to determine the correct response. According to previous studies, the P3 is reduced in clinical groups such as those with schizophrenia and ADHD (25, 26). Longer P3 latency elicited by incongruent trials implies the increased stimulus evaluation or categorization time (13, 27). These behavioral and neural markers of conflict control can capture subtle differences in cognitive processing and serve as ideal indicators for identifying cognitive deficits in trait anxiety.

Although dysfunctional forms of cognitive processing in trait anxiety have been well evidenced, more extensive studies are necessary, because findings related to emotional regulation mainly restricted to persons diagnosed with GAD. Recent research in non-clinical anxiety revealed that there are different components of anxiety-related cognitive control, which have different clinical implications (29). However, so far, few studies have directly examined the mechanisms responsible for the effect of trait anxiety on cognitive control based on clinical and non-clinical individuals simultaneously. Therefore, in this study, healthy individuals with low levels of trait anxiety [low trait anxiety (LTA)], healthy individuals with high levels of trait anxiety (HTA), and trait anxious patients with a diagnosis of GAD were recruited and emotional flanker task was adopted to examine two issues (1): how trait anxiety affects processing efficiency and performance effectiveness for HTA and GAD individuals separately? (2) Whether trait anxiety inevitably elicits conflict maladaptation. Based on empirical and theoretical evidence, we hypothesize that: (1) relative to LTA individuals, HTA ones should display at a level-standard performance effectiveness at the expense of processing efficiency, while GAD patients have shortfalls in both performance effectiveness and processing efficiency (2). For both HTA and GAD individuals, trait anxiety will impair conflict detection and conflict resolution, thereby leading to conflict maladaptation.

## Materials and Methods

### Participants

This study was approved by the ethics committee of Third Military Medical University of China. The total sample was consisted of three subgroups: LTA, HTA, and GAD. All of them provided written consent after a detailed explanation of the study aims and procedures. Participants received 50 RMB for their time.

Initially, through announcements (intranet, Internet, and local poster), 1,539 healthy persons aged from 16 to 45 were recruited to take part in a mass screening by assessing their levels of trait anxiety (30, 31). Subsequently, persons in the lower 27% of the trait anxiety distribution [State-Trait Anxiety Inventory (STAI_T) ≤ 33] were assigned to the LTA group, and the ones in the higher 27% of the trait anxiety distribution (STAI_T ≥ 40) were assigned to the HTA group.

Individuals who were willing to take part in the following experiments were asked to complete the Center for Epidemiologic Studies Depression Scale (CES-D) (32), fill basic personal information, and report the past history of disease. The inclusion criteria in the present study for the normal participants were as follows: (1) no less than 9 years of education; (2) normal or corrected-to-normal vision; (3) provided informed consent to take part in the present research; (4) no evidence of substance abuse or dependence in the past 3 months; and (5) no mental and cognitive disorders or brain injury.

High trait anxious patients diagnosed with GAD were recruited from the outpatient clinic of Xinqiao Hospital and Daping Hospital of Chongqing, China. Prior to participation, they were diagnosed by two licensed clinical psychologists. Diagnoses were confirmed using the Chinese Version of Mini-International Neuropsychiatric Inventory (33, 34). Then, they completed measures of trait anxiety, depression, and detailed information regarding the inclusion criteria. The inclusion criteria for the GAD participants were as follows: (1) aged between 16 and 45 years; (2) no less than 9 years of education; (3) normal or corrected-to-normal vision; (4) with heightened level of trait anxiety (STAI_T ≥ 40); (5) no evidence of substance abuse or dependence in the past 3 months; (6) no history of schizophrenia, bipolar disorder, organic mental disorder or brain injury; and (7) no treatment of electric shock, repetitive transcranial magnetic stimulation, deep brain electrical stimulation, or other electromagnetic techniques in the past 6 months. Study enrollment included 29 LTA individuals, 37 HTA individuals, and 23 patients with GAD.

### Materials and Tasks

#### Self-Report Measures

The trait subscale of Spielberger’s STAI_T (30) was used to measure the level of trait anxiety. This subscale consists of 20 items that can indicate individuals’ tendency to perceive stressful situations as dangerous or threatening. Answers were given on a 4-point Likert scale. This measure has adequate psychometric properties. Internal consistency was Cronbach’s alpha = 0.950 for STAI_T in the current study. The CES-D (32) was adopted to measure the level of depression. The CES-D is a self-report scale specifically designed for epidemiological studies to assess the presence of clinical and non-clinical symptoms of depression in the general population. The CESD consists of 20 items with adequate psychometric properties (35). Internal consistency for the sample was Cronbach’s alpha = 0.953 for CES-D in this study.

#### Apparatus and Stimuli

All stimuli were presented on a 17-inch Lenovo CRT monitor with a resolution of 1,024 × 768 pixels. E-Prime 2.0 Software Package was used to run the emotional flanker task. Participants were seated about 70 cm from the computer screen and performed emotional flanker task.

#### Emotional Flanker Task

**Figure 1:**
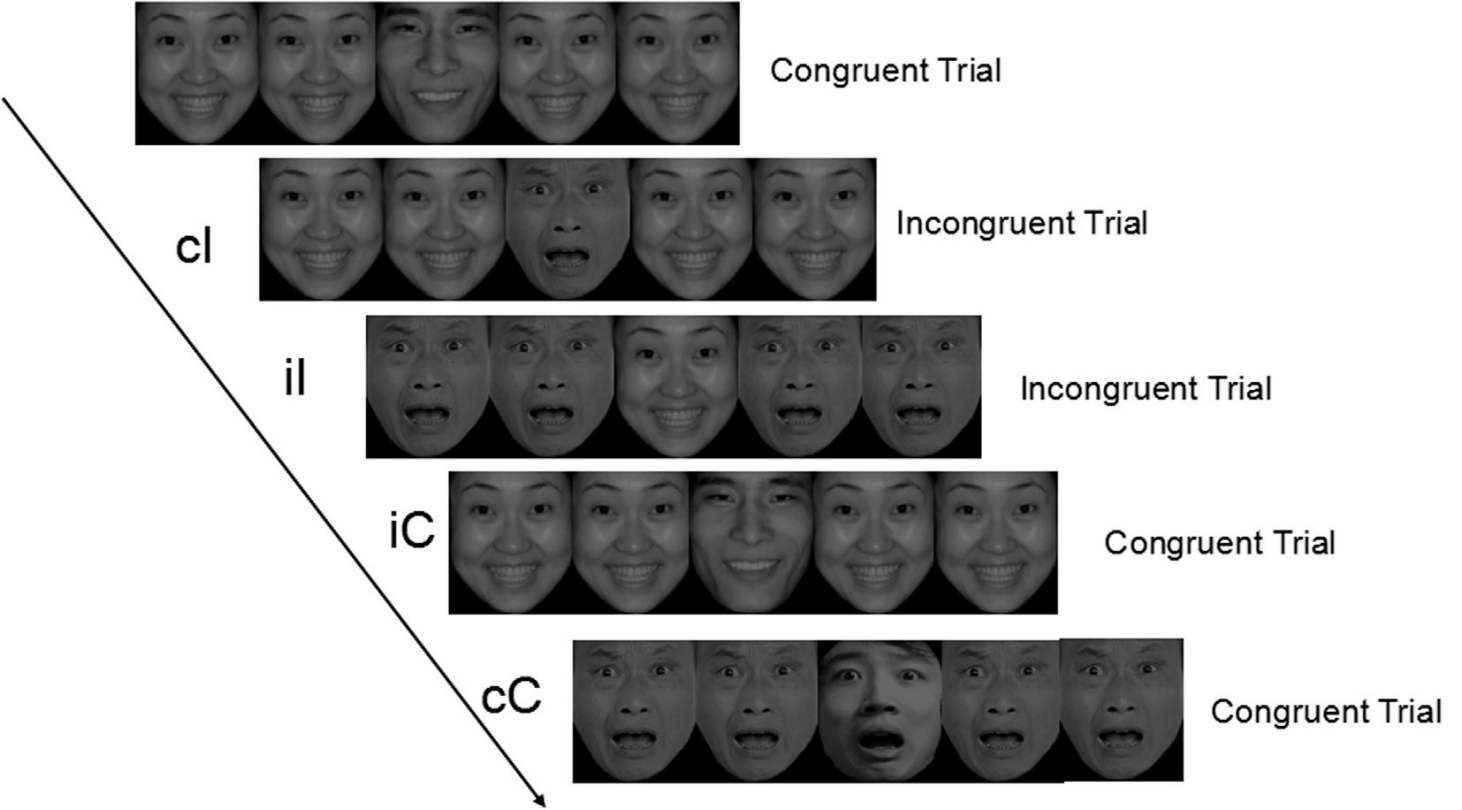
Graphical representation of trials and experimental conditions. cC, congruent trials preceded by congruent trials; cI, incongruent trials preceded by congruent trials; iI, incongruent trials preceded by incongruent trials; iC, congruent trials preceded by incongruent trials.

Photos of 24 different people (12 female, 12 male) showing happy or angry emotional expressions (the ratio was 1:1) were chosen from the standardized native Chinese Affective Picture System (CAPS) (36). On each trial, the target face (2.05° × 2.37°) was surrounded by two flanker faces that owned either congruent or incongruent emotion with the target on right and left sides. Target and flankers always appeared at the same positions on the black background. Participants were instructed to respond to the emotion of the central face by pressing “f” button for happy faces and “j” for angry ones while ignoring the flanker faces. Participants were encouraged to respond to the stimuli as quickly and accurately as possible. There was one practice block and four experimental blocks. The task consisted of 25 practice trials and 196 experimental trials. Four photos used in the practice block did not disappear in the following experimental blocks. Each trial began with a fixation cross displayed on the center of the screen for 500 ms. The fixation cross was then replaced by a target face with two flankers located at the left and right of each target. Stimuli were presented in a pseudorandom order and remained on the screen until the participant responded. A varying interstimulus interval was set between 800 and 1,500 ms. There was a break between each block. Completion of the experiment required about 15 min. The schematic experimental procedure of the emotional flanker task is illustrated in Figure 1.

The flanker task comprised four types of experimental conditions according to the match between the current and the previous trial: congruent—Congruent (cC), congruent—Incongruent (cI), incongruent—Incongruent (iI), and incongruent—Congruent (iC). According to Nieuwenhuis et al. (37), index of conflict adaptation effect (CAE) on RT (CAE_RT_) can be computed as follows: CAE_RT_ = (RT_cI_ − RT_cC_) − (RT_iI_ − RT_iC_). Formulas used to calculate CAEs on error rates, N2 and P3 components are similar to the aforementioned one.

#### ERP Recording and Analysis

The experiment was conducted in a dimly lit and electrically shielded room. Each participant was asked to sit still and minimize blinks and movements during electroencephalography (EEG) recording. A high-density EEG recording was acquired with a QuickAmp amplifier using 64 Ag/AgCl electrodes. All electrodes were referenced on line to the Cz position with a ground electrode on AFz. Horizontal and vertical electrooculogram signals were recorded with four bipolar electrodes placed on the outer canthus of each eye as well as above and below the right eye. The EEG activity was amplified using 0.01–100 Hz band-passed filters and sampled at 1,000 Hz. Impedance was kept below 5 kΩ for all electrodes.

MATLAB 2013b (MathWorks, USA) and the EEGLAB13.4.4b toolbox (38) were used to conduct offline EEG analyses. For offline analysis, the EEG data were filtered using a bandpass between 0.5 and 30 Hz and re-referenced to the average of the two mastoids. Then, the data were segmented into epochs ranging from 200 ms prestimulus to 700 ms post-stimulus. Baseline correction was performed using the prestimulus interval. Epochs were rejected if the voltage deviated more than 5 SD values of the probability distribution. Finally, the runica function of EEGLAB was used to perform independent components (ICs). ICs identified as muscle activity, eye movements, eye blinks, or other types of noise were removed from the EEG signal.

The mean amplitudes were calculated from latency windows of ±10 ms around the maximum peaks latencies identified from the mean global field power (28) that were obtained including all participants and all conditions for each type of stimulus. Two late ERP components were used to test predictions from the conflict monitoring model: the frontal N2 and the central P3. The N2 component was measured as the most negative local amplitude between 200 and 300 ms post-stimulus on the average of five fronto-central electrodes (Fz, FCz, FC1, FC2, and Cz). The P3 component was measured as the most positive local amplitude between 300 and 500 ms post-stimulus on the average of five centro-parietal electrodes (Cz, CPz, CP1, CP2, and Pz).

### Data Analysis

Outliers were removed in keeping with recommendations from Ratcliff (39). Participants with mean accuracy less than 75% were excluded from analysis, which resulted in the exclusion of one participant from the LTA group, two participants from the HTA group, and two participants from the GAD group. Trials that involved incorrect responses and RTs exceeding 3 SD from mean RTs (1.63%) were eliminated from the data. Besides that, 6 participants were excluded due to their EEG data loss resulting from machine fault, and 7 participants were excluded because of too few effective ERP epochs (no less than 20 each condition). Finally, there are 84 valid participants for the behavioral data and 66 valid participants for the ERP data.

IBM SPSS software V18 (IBM Corp., Armonk, NY, USA) was used for further statistical analyses. Controlling for three sociodemographic variables (age, gender, and educational level), a series of 3 (group: LTA, HTA, and GAD) × 2 (trial type: congruent and incongruent) repeated measures analysis of variance (ANOVA) were conducted on mean RTs and error rates as well as on the latencies and amplitudes of N2 and P3 components in order to assess the main effects and interactions. The indexes of CAE on behavior and ERP data were calculated separately according to the calculation formula of CAE. Subsequently, one-way ANOVA was carried out to examine the study group difference. According to the Greenhouse–Geisser method, the degrees of freedom for all repeated measures ANOVAs were corrected. The correlations between trait anxiety and RTs, error rates, and ERP data were also examined.

Using Lilliefors significance correction, Kolmogorov–Smirnov statistic analysis verified that behavioral results and ERP data approximated normal distribution (for complete sample or each group separately, *P*s = 0.239–0.101 > 0.05). For all analyses in this study, the significance level was set at *P* < 0.05. Results are presented as mean ± *SD*.

## Results

### Demographics and Self-Report Data

Table 1 shows participant characteristics. The LTA, HTA, and GAD groups did not significantly differ in gender, age or education which indicated that these groups well matched with respect to demographic variables. As expected, there were significant group differences in trait anxiety and depression (*P*s < 0.001). Specifically, participants with GAD had significantly higher levels of STAI-T and CES-D compared to those of LTA and HTA groups (*Ps* < 0.001), while participants in the HTA group had significantly greater STAI-T and CES-D scores than those of the LTA group (*P* < 0.001).

**Table 1:** Demographic and questionnaire data for participants (mean ± *SD*).

		Low trait anxiety (29)	High trait anxiety (36)	Generalized anxiety disorder (21)	*P*
Education	Less than high school	0	1 (2.9%)	3 (14.3%)	0.100
	Completed high school	25 (89.3%)	24 (68.6%)	7 (33.3%)	
	Junior college or Bachelor’s degree	3 (10.7%)	6 (17.1%)	10 (47.6%)	
	Graduate	0	4 (11.4%)	1 (4.8%)	

% Female		21.43	19.44	42.86	0.189
Age		23.85 ± 4.10	24.11 ± 6.16	27.19 ± 7.11	0.161
STAI-T		28.32 ± 3.43	46.49 ± 5.11	57.86 ± 8.31	<0.001
CES-D		1.25 ± 1.71	13.51 ± 9.92	31.14 ± 13.67	<0.001

### Behavioral Results

Descriptives of mean RTs and error rates in each condition in the emotional flanker task are presented in Table 2. Controlling for three sociodemographic variables, a two-way repeated measures ANOVA on mean RTs was conducted with group as the between-subjects variable and trial type as the within-subjects variable. Neither the interaction of group and trial type [*F*(2,81) = 0.251, *P* = 0.779, $\eta_{P}^{2}=0.006$], nor the main effect of trial type [*F*(1,81) = 0.467, *P* = 0.496, $\eta_{P}^{2}=0.006$] reached statistical significance. Nevertheless, a significant main effect of group was found [*F*(2,81) = 51.299, *P* < 0.001, $\eta_{P}^{2}=0.568$]. *Post hoc* comparisons between groups showed that RTs of the GAD group (928.84 ± 140.86) were significantly longer than those of the LTA (630.31 ± 82.83, *P* < 0.001) and HTA groups (655.06 ± 93.18, *P* < 0.001). No significant difference was found between the LTA and HTA groups (*P* = 0.380).

**Table 2:** Mean RTs and error rates in emotional Flanker task (mean ± SD).

	LTA	HTA	GAD
Congruent (RT)	637.41 ± 88.88	656.75 ± 92.42	930.40 ± 149.09^†^^‡^
Incongruent (RT)	627.92 ± 83.36	653.35 ± 94.82	909.84 ± 150.00^†^^‡^
Congruent (error rate)	1.49 ± 1.83	1.31 ± 1.96	2.13 ± 2.04^‡^
Incongruent (error rate)	1.30 ± 2.04	1.43 ± 2.01	2.88 ± 2.42^†^^‡^
cC (RT)	635.56 ± 89.43	659.35 ± 97.20	937.45 ± 150.72^†^^‡^
cI (RT)	629.83 ± 86.54	650.85 ± 99.01	926.18 ± 158.99^†^^‡^
iC (RT)	639.19 ± 89.89	654.23 ± 91.10	923.76 ± 153.92^†^^‡^
iI (RT)	625.89 ± 82.75	656.02 ± 94.96	927.78 ± 135.15^†^^‡^
Conflict adaptation effect (CAE) (RT)	7.57 ± 30.30	−10.29 ± 46.17	−15.28 ± 86.76
cC (error rate)	1.04 ± 1.65	1.43 ± 2.46	1.98 ± 2.33
cI (error rate)	1.48 ± 2.83	1.31 ± 1.83	2.68 ± 2.56^†^^‡^
iC (error rate)	1.93 ± 2.66	1.19 ± 2.16	2.28 ± 2.79^‡^
iI (error rate)	1.12 ± 1.84	1.55 ± 2.44	2.90 ± 3.20^†^^‡^
CAE (error rate)	1.26 ± 3.60	−0.48 ± 2.67	−0.10 ± 4.91

*LTA, low trait anxiety; HTA, high trait anxiety; GAD, generalized anxiety disorder; cC, congruent trials preceded by congruent trials; cI, incongruent trials preceded by congruent trials; iI, incongruent trials preceded by incongruent trials; iC, congruent trials preceded by incongruent trials*.

*^†^There was a statistically significant difference between this group and the LTA group (*P* < 0.05)*.

*^‡^There was a statistically significant difference between this group and the HTA group (*P* < 0.05)*.

*Sociodemographic variables were included as covariates for all analyses*.

Similar results were obtained by a two-way repeated measures ANOVA on error rates. Both the interaction of group and trial type [*F*(2,81) = 1.594, *P* = 0.210, $\eta_{P}^{2}=0.039$] and the main effect of trial type [*F*(1,81) = 0.366, *P* = 0.547, $\eta_{P}^{2}=0.005$] far from significance. However, the main effect of group was found to be significant [*F*(2,81) = 3.904, *P* = 0.024, $\eta_{P}^{2}=0.091$]. *Post hoc* multiple comparisons showed that error rates for the GAD group (2.51 ± 1.95) were significantly larger than those for the LTA (1.38 ± 1.68, *P* = 0.023) and HTA groups (1.37 ± 1.85, *P* = 0.009), and no significant difference was observed between the LTA and HTA groups (*P* = 0.795).

Also, the indexes of CAE on RTs and error rates were calculated. After controlling for three sociodemographic variables, a one-way ANOVA was performed to check the group difference in CAE. There was no significant difference among these three groups in CAE of RTs, *F*(2,81) = 0.903, *P* = 0.410. A similar result was observed in CAE of error rates, *F*(2,81) = 1.305, *P* = 0.277.

### ERP Data

**Figure 2:**
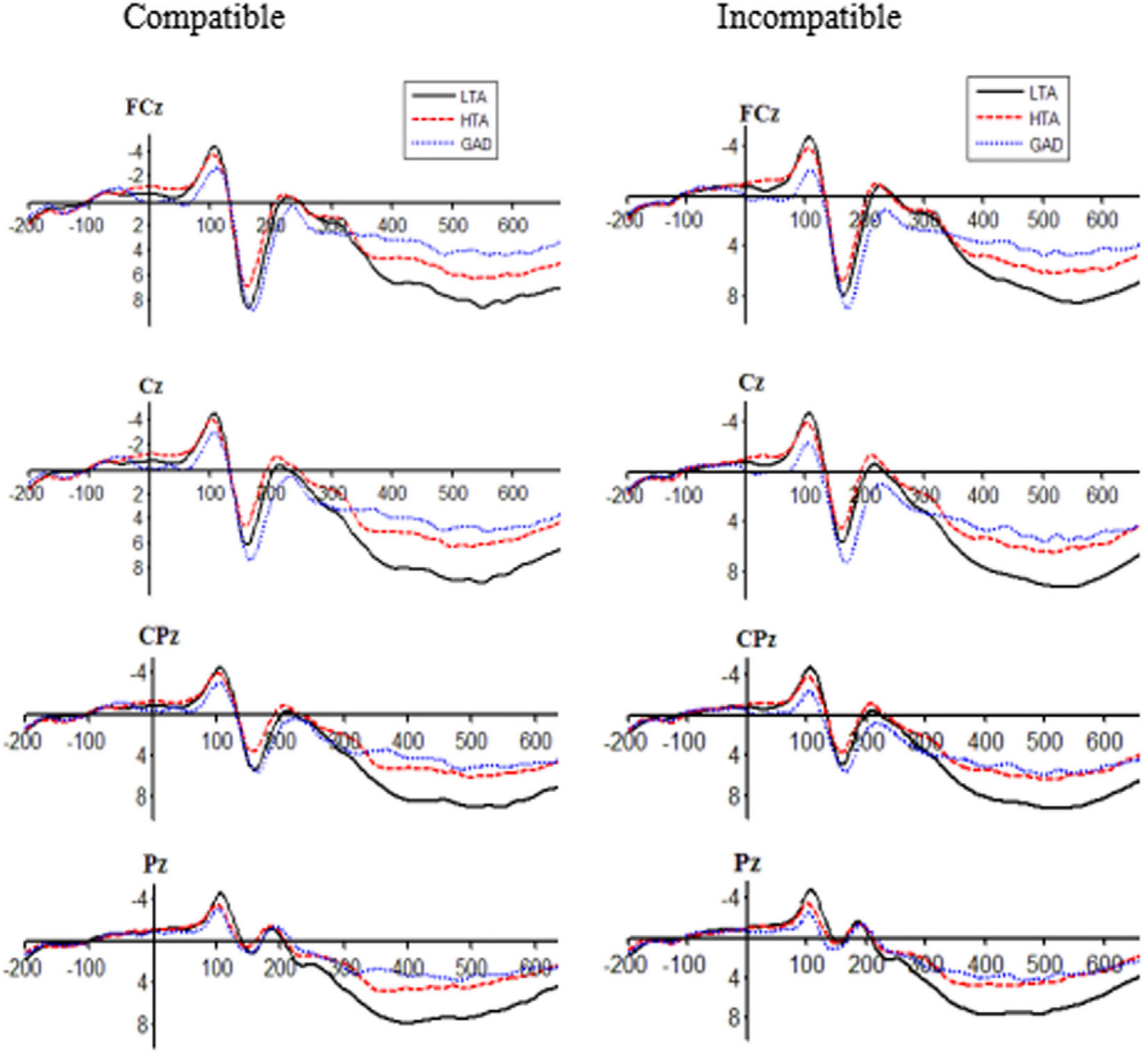
Stimulus-locked event-related potentials for the low trait anxiety (LTA), high trait anxiety (HTA), and generalized anxiety disorder (GAD) groups in compatible and incompatible conditions at FCz, Cz, CPz, and Pz sites.

Figure 2 shows stimulus-locked ERPs for compatible and incompatible stimuli from midline electrode sites (FCz, Cz, CPz, and Pz). Peak amplitudes and latencies for N2 and P3 components are listed in Table 3.

**Table 3:** Amplitude (microvolts) and latency (milliseconds) of event-related potential components in emotional Flanker task (mean ± SD).

	LTA (*n* = 23)	HTA (*n* = 24)	GAD (*n* = 19)
Compatible_N2 amplitude	−1.66 ± 3.22	−2.29 ± 3.69	−0.97 ± 2.87
Incompatible_N2 amplitude	−1.78 ± 3.65	−2.58 ± 3.50	−0.29 ± 2.85
Compatible_N2 latency	221.60 ± 20.67	218.87 ± 19.10	226.79 ± 20.88
Incompatible_N2 latency	216.82 ± 19.73	216.11 ± 19.74	229.58 ± 20.42

Compatible_P3 amplitude	10.09 ± 3.54	6.97 ± 2.80	6.31 ± 2.56
Incompatible_P3 amplitude	9.79 ± 3.33	7.05 ± 2.84	6.73 ± 2.34
Compatible_P3 latency	404.53 ± 67.42	423.04 ± 54.59	432.75 ± 50.95
Incompatible_P3 latency	401.75 ± 54.65	420.54 ± 44.62	434.57 ± 48.45

*LTA, low trait anxiety; HTA, high trait anxiety; GAD, generalized anxiety disorder*.

After controlling for three sociodemographic variables, a two-way repeated measures ANOVA on N2 amplitude showed no significant main effect of group [*F*(2,63) = 1.082, *P* = 0.346, $\eta_{P}^{2}=0.035$] or trial type [*F*(1,63) = 0.254, *P* = 0.616, $\eta_{P}^{2}=0.004$]. However, the interaction effect of group and trial type yielded a clear tendency to significance [*F*(2,63) = 2.898, *P* = 0.063, $\eta_{P}^{2}=0.008$]. After controlling for sociodemographic variables, multiple comparisons showed that HTA individuals had more negative N2 peak amplitudes (−2.58 ± 3.50) relative to GAD patients (−0.29 ± 2.85, *P* = 0.039) for incompatible trials. N2 amplitude of the LTA group (−1.66 ± 3.22) did not differ from those of the HTA and GAD groups (*P* = 0.428, *P* = 0.184, respectively). No group difference was observed for compatible trials on N2 amplitude (*P* > 0.05). For the N2 latency, main effects of group [*F*(2,63) = 2.143, *P* = 0.126, $\eta_{P}^{2}=0.067$] and trial type [*F*(1,63) = 1.392, *P* = 0.243, $\eta_{P}^{2}=0.023$], and the interaction effect [*F*(2,63) = 2.194, *P* = 0.144, $\eta_{P}^{2}=0.035$] were not statistically significant.

Likewise, P3 amplitude and latency were separately subjected to repeated measures ANOVAs. For the P3 amplitude, after controlling for sociodemographic covariates, neither the interaction effect [*F*(2,63) = 2.341, *P* = 0.105, $\eta_{P}^{2}=0.072$] nor the main effect of trial type [*F*(1,63) = 0.001, *P* = 0.972, $\eta_{P}^{2}=0.001$] reached significance. However, we found a significant main effect of group [*F*(2,63) = 8.268, *P* = 0.001, $\eta_{P}^{2}=0.216$] such that the LTA group exhibited more positive P3 amplitudes (9.94 ± 0.60) than the HTA and GAD groups (amplitude = 7.01 ± 0.59, *P* = 0.001; amplitude = 6.52 ± 0.66, *P* < 0.001, respectively). No significant difference was observed between the HTA and GAD groups (*P* = 0.553). For the P3 latency, it was showed that the main effect of group [*F*(2,63) = 0.182, *P* = 0.672, $\eta_{P}^{2}=0.003$], the main effect of trial type [*F*(1,63) = 0.540, *P* = 0.468, $\eta_{P}^{2}=0.009$], and the interaction effect [*F*(2,63) = 0.348, *P* = 0.708, $\eta_{P}^{2}=0.011$] were far from statistical significance.

We also calculated the indexes of CAE on N2 and P3. To check whether the CAE was influenced by trait anxiety, one-way ANOVAs were separately performed for N2 and P3 amplitudes and latencies after controlling for age, gender, and educational level. There was a significant difference in CAE on the N2 amplitude across study groups [*F*(2,63) = 4.598, *P* = 0.014, $\eta_{P}^{2}=0.133$]. *Post hoc* multiple comparisons showed that the LTA group had a larger index of CAE on the N2 amplitude (0.57 ± 2.22) relative to the HTA (−1.34 ± 2.85, *P* = 0.012) and GAD groups (−1.90 ± 2.57, *P* = 0.011). By contrast, indexes of CAE on N2 latency, P3 amplitude, and P3 latency did not vary across study groups (*P* = 0.228, *P* = 0.537, *P* = 0.370, respectively).

### Correlation Analyses

To examine the relationship between trait anxiety scores and behavior data, N2 and P3 components across a range of symptom severity, we included all participants in correlation analyses. Scores of trait anxiety were significantly related to RTs, Pearson’s *r*(84) = 0.602, *P* < 0.001. A similar result was obtained between scores of trait anxiety and error rates *r*(84) = 0.226, *P* = 0.038. On the other hand, trait anxiety was not related with N2 component [*r*(66) = 0.126, *P* = 0.314 for N2 amplitude; *r*(66) = 0.175, *P* = 0.158 for N2 latency]. HTA is associated with decreased P3 amplitude, *r*(66) = −0.465, *P* < 0.001, but not for trait anxiety and P3 latency, *r*(66) = 0.209, *P* = 0.091.

Associations between trait anxiety scores and the indexes of CAE for behavioral results and ERP data were also assessed by correlation analyses. Higher trait anxiety scores were associated with smaller CAE on RTs, Pearson’s *r*(84) = −0.219, *P* = 0.046, but not on error rates, *r*(84) = 0.165, *P* = 0.134. In addition, there was a significant correlation between trait anxiety and CAE on N2 amplitude, *r*(66) = −0.356, *P* = 0.003, indicating decreased conflict adaptation for individuals with higher trait anxiety. However, no significant correlation was found between trait anxiety and the indexes of CAE on N2 latency, P3 amplitude, and P3 latency (*P* = 0.259, *P* = 0.328, *P* = 0.552, respectively).

## Discussion

Using event-related brain potentials, we examined cognitive control in an emotional flanker task among non-clinical individuals with LTA, non-clinical individuals with HTA, and patients with GAD. The behavioral results revealed that GAD patients had prolonged response times and increased error rates in emotional flanker task as compared to LTA and HTA individuals. ERPs data demonstrated that in incompatible trials, HTA individuals exhibited a larger N2 amplitude relative to GAD individuals. It was also suggested that HTA and GAD individuals had a smaller P3 amplitude than LTA individuals. Furthermore, CAE contrasts among three study groups showed that LTA individuals owned a better ability in conflict adaptation than the other two groups on N2 amplitude.

High trait anxiety individuals did not reveal prolonged response time and increased behavioral errors, but showed a trend of increased N2 amplitude, reflecting compensatory activation to conflict stimuli. Since a larger N2 amplitude may reflect greater resources being devoted to action monitoring (40, 41), our results suggested that individuals in the HTA group maintained intact work performance with low anxious individuals by recruiting greater cognitive resources and giving more effort. Therefore, our hypothesis that trait anxiety impaired processing efficiency rather than performance effectiveness for individuals with HTA was approved. Similar results were also obtained in the stop-signal task by Savostyanov et al. (42). Greater EEG desynchronization was found in anxious individuals, indicating that more processing effort and resource allocation were required to inhibit a motor response. Coincidentally, ACT argues that in some cases people with high anxiety do not show greater evidence of disrupted attentional control behaviorally, but more cognitive resource was required to perform at a level-standard with low anxious individuals (1, 2). Compared to the LTA group, HTA individuals exhibited weaker P3 components. On account of implications of N2 and P3 components, it was the first time to discover that HTA individuals had a high vigilance to the emotional conflict; however, they showed a deficit in emotion regulatory capability. That is, HTA individuals appeared to have an overactive conflict detection process but poor ability to conflict resolution, which is not inconsistent with our study hypothesis.

By contrast, patients with GAD are associated with deficits in cognitive efficiency with prolonged response times and increased error rates. This is in high agreement with previous studies. For example, in an *N*-back task, Balderston et al. reported that GAD patients showed an overall impairment in both accuracy and reaction time compared to controls (43). Similarly, another empirical study found that clinician-rated anxiety severity predicted slower and less accurate Stroop performance over and above the effect of GAD diagnosis (44). At the neural level, compared to HTA individuals, GAD patients revealed decreased N2 components, while compared to LTA individuals, GAD patients exhibited weaker P3 components. These results suggested that GAD patients could not utilize their limited cognitive resources to achieve the desirable performance outcome. Our results fit better with previous findings that GAD patients showed less activation in the dorsolateral prefrontal cortex, a region critical for cognitive control (43). Therefore, our hypothesis that trait anxiety impaired their processing efficiency and performance effectiveness for GAD patients was proved. Meanwhile, GAD was related to a poor ability to conflict detection and conflict resolution.

Our study found that the cognitive and neural processes implicated in conflict adaptation were altered in the HTA and GAD groups. At the neural level, a significant group difference was found in the index of CAE on N2 amplitude, indicating that the HTA and GAD groups revealed obvious perturbations in emotional conflict adaptation as compared to the LTA group. Furthermore, both the HTA and GAD groups exhibited decreased P3 amplitudes. These results demonstrated that the ability for conflict resolution can be seriously impaired by trait anxiety, thereby resulting in their difficulty in conflict adaptation. It agrees well with a most recent study which found that the P3 amplitude of target stimuli was reduced due to the influence of distraction on anxious individuals (45). This study also corroborates neuroimaging findings by demonstrating that GAD is associated with attenuated response to conflict, which results in impaired top-down control and emotional dysregulation (11).

These results are of great significance for the study of psychiatric diseases. It has been widely assumed that cognitive control studies in subclinical analog samples can be generalized to the corresponding clinical disorder (28). Our findings imply that the pattern of impaired cognitive control, as reported in the high trait anxious sample from normal populations, cannot be directly generalizable to clinical anxiety. We did find significant differences between the HTA and GAD groups. Specifically, different from HTA individuals who had intact performance effectiveness, GAD patients showed impaired cognitive function with prolonged response and poor accuracy in the emotional conflict task. Besides, HTA individuals recruited more cognitive resources to monitor conflict information than GAD patients. Nevertheless, they still have some consistent features in conflict control. Both HTA individuals and GAD patients had impaired processing efficiency and poor abilities to conflict resolution due to their failures in conflict adaptation and decreased P3 amplitudes. These results can deepen and extend our understanding that GAD is associated with a severely impaired cognitive control, while HTA individuals appear to have a moderately impaired cognitive control.

Taken together, the current findings based on clinical and non-clinical individuals shed light on the essential relationship between trait anxiety and cognitive control. In addition, our results distinguish the features of HTA individuals and GAD patients in emotional conflict control. Nevertheless, similar to other studies, our result suffers from a number of limitations. First, the sample size is relatively small, and therefore, it is insufficient to detect significant among-group differences in conflict adaptation on N2 amplitude. Nevertheless, the magnitude of the trend makes our finding clinically meaningful. These results should be further verified. Second, the results were based on the emotional flanker task. According to a most recent study, anxious individuals preferentially allocate attention to emotional distractors who subsequently exhibit poorer cognitive performance (46). Etkin et al. also asserted that abnormal conflict processing for the clinical patients diagnosed with GAD only manifests in the regulation of emotional conflict, rather than non-emotional conflict (11). Non-emotional flanker task (e.g., arrows) may not elicit impaired cognitive control for the HTA and GAD groups. Therefore, our results need to be replicated and verified in non-emotional flanker stimuli. Third, state anxiety and trait anxiety are highly correlated. According to previous studies, they both have adverse effects on cognitive function (1, 2). However, state anxiety was not assessed in this study. Accordingly, state anxiety and trait anxiety need to be assessed simultaneously in future studies.

Despite these limitations, several key implications can be drawn to better understand the relationship between trait anxiety and conflict control in task. The results in the present study revealed that HTA individuals exhibited comparable performance effectiveness to LTA individuals at the expense of processing efficiency, while GAD patients had shortfalls in both performance effectiveness and processing efficiency. Moreover, HTA individuals revealed poor abilities for conflict resolution rather than for conflict detection, while individuals diagnosed with GAD had impaired conflict detection and conflict resolution functions. Our research provides a powerful support for the viewpoint that trait anxiety can elicit conflict adaptation impairments and suggest that trait anxiety is a predominant factor at the onset of and in the maintenance of GAD. Therefore, trait anxiety reducing strategies may provide significant therapeutic gains.

## End Notes

The following images from the Chinese affective picture system were used in this study in the experimental blocks: angry: AF3, AF5, AF9, AF15, AF23, AM1, AM7, AM20, AM24, and AM33; happy: HF11, HF50, HF115, HF119, HF122, HM10, HM92, HM93, HM94, and HM97.

## Ethics Statement

The study was approved by the institutional review board of Third Military Medical University. The research protocol was carried out in accordance with the recommendations of the principles of Declaration of Helsinki. Written informed consent was obtained from each participant.

## Author Contributions

YY, LP, BL, and ML designed research; YY, CJ, HX, QY, and WX performed experiments; YY, JL, and YX analyzed data; YY, CJ, FL, and ML wrote and revised the paper.

## Conflict of Interest Statement

The authors declare that the research was conducted in the absence of any commercial or financial relationships that could be construed as a potential conflict of interest. The reviewer RS and handling Editor declared their shared affiliation.
